# Supplementary material for: Longitudinal Live Imaging Derived 4D Hemodynamics and Dynamic Tissue Mechanics Across Outflow Tract Morphogenesis
Source: bioRxiv. 2025 Aug 30:2025.08.26.672464. Preprint. [Version 1] doi: 10.1101/2025.08.26.672464 (PMC12407944; doi:10.1101/2025.08.26.672464)
Supplement: Supplement 1 [file media-1.zip › Supplemental Materials/supplemental files_longitudinal_4D_BiorXiv.pdf]

Supplemental Figures

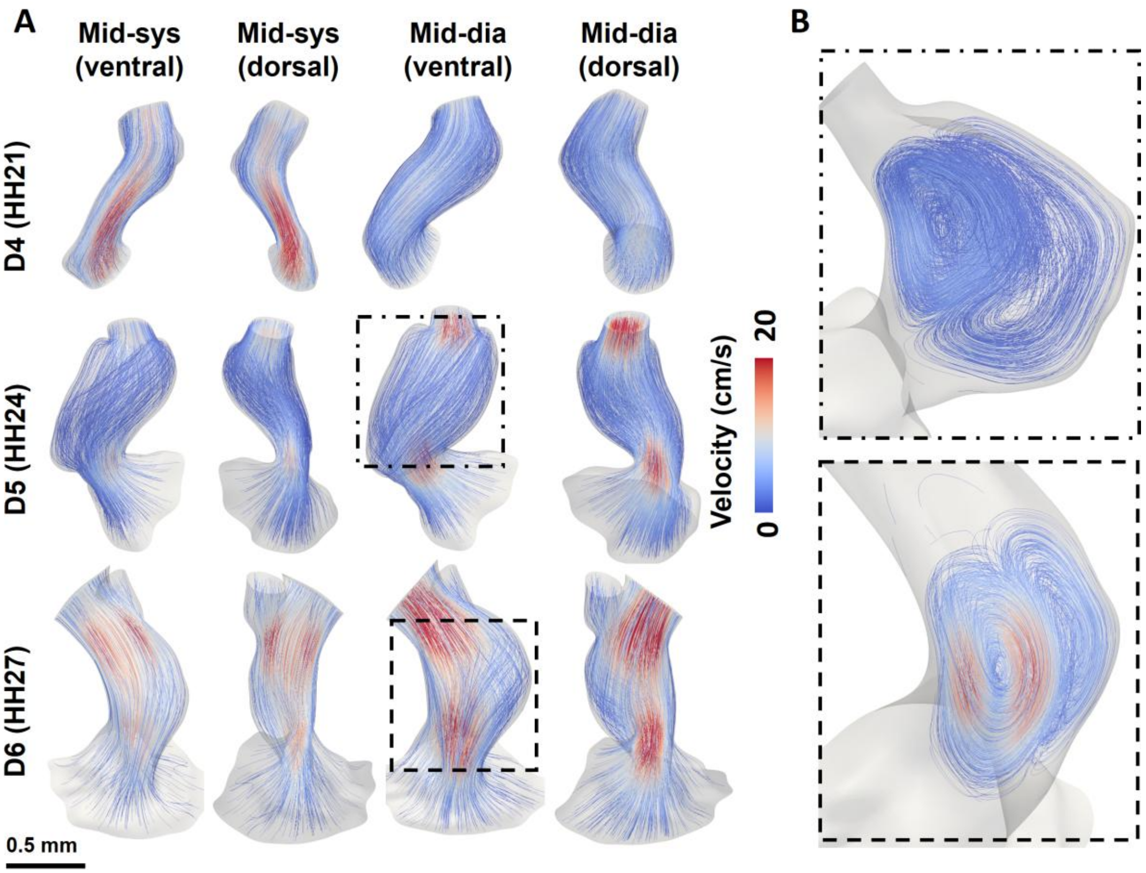

Figure S1: Time-varying streamline within OFT lumen. A) Time- and space-varying streamlines contoured with velocity magnitudes at different phases of cardiac cycle over development. Figures show the 3D renderings of the velocity streamlines within the dynamic models of Embryo 1 at different developmental stages at mid-systole and mid-diastole. Higher velocity magnitudes were observed at the proximal cushions and distal cushions during systole after cushion opening. **B)** Vortex flow in the intermediate OFT region during cushion shut.

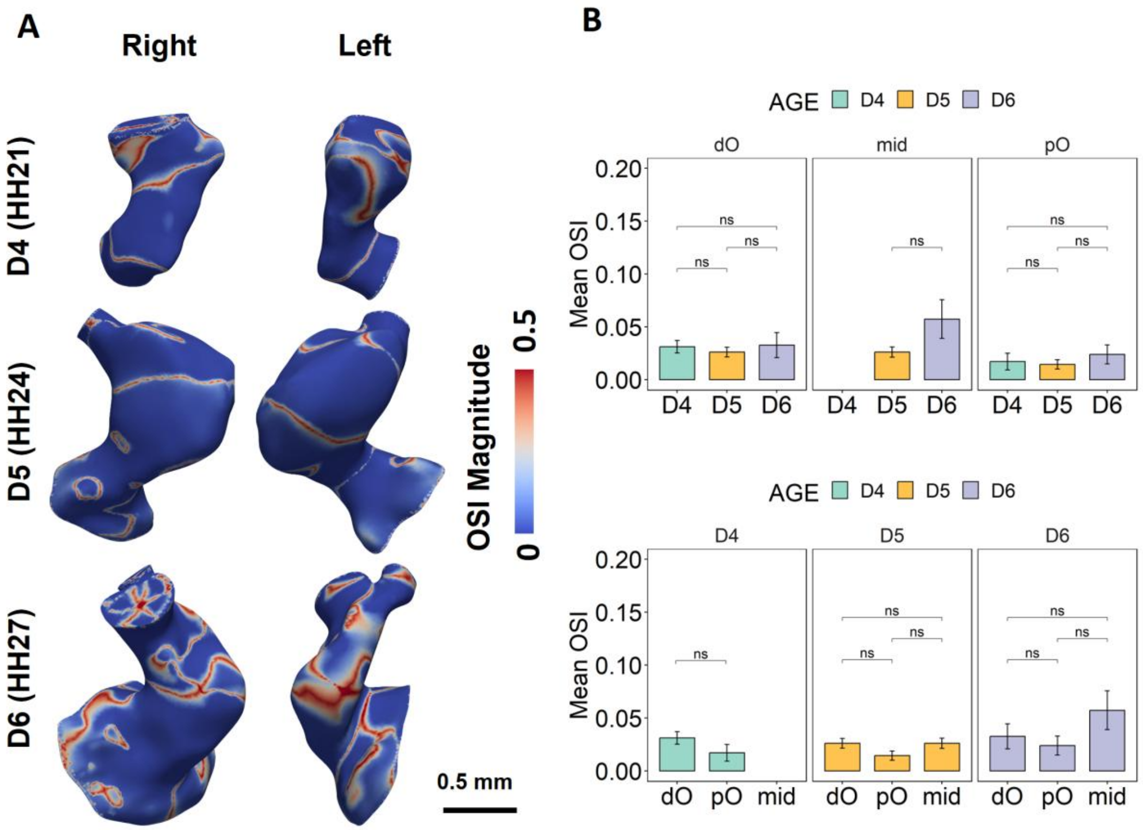

Figure S2: Oscillatory shear index in the OFT lumen across development. A) Models of Embryo 1 at the segmentation time frame were used to show the spatial variation of OSI across development. Higher OSI in the intermediate OFT region at HH24 and HH27. **B)** No difference in regional mean OSI magnitudes over time or among regions.

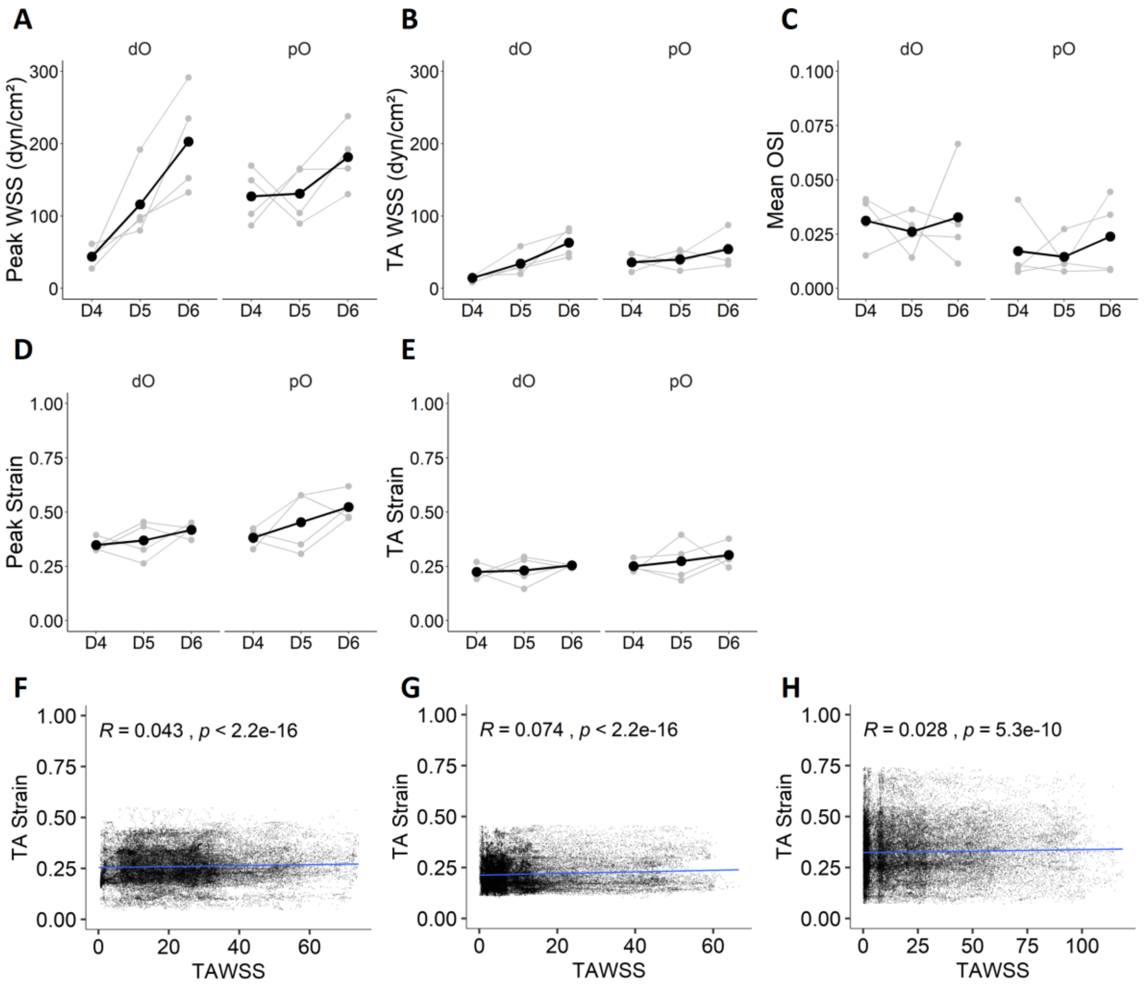

Figure S3: Developmental trends of A) peak WSS, B) time-averaged WSS, C) OSI, D) peak strain, and E) time-averaged strain in 4 embryos. Data points from the same embryo were linked with black lines across development. **No spatial correlations between strain features and WSS at F) HH21, G) HH24, and H) HH27.** Data points represent strain and TAWSS magnitudes of the same elements.

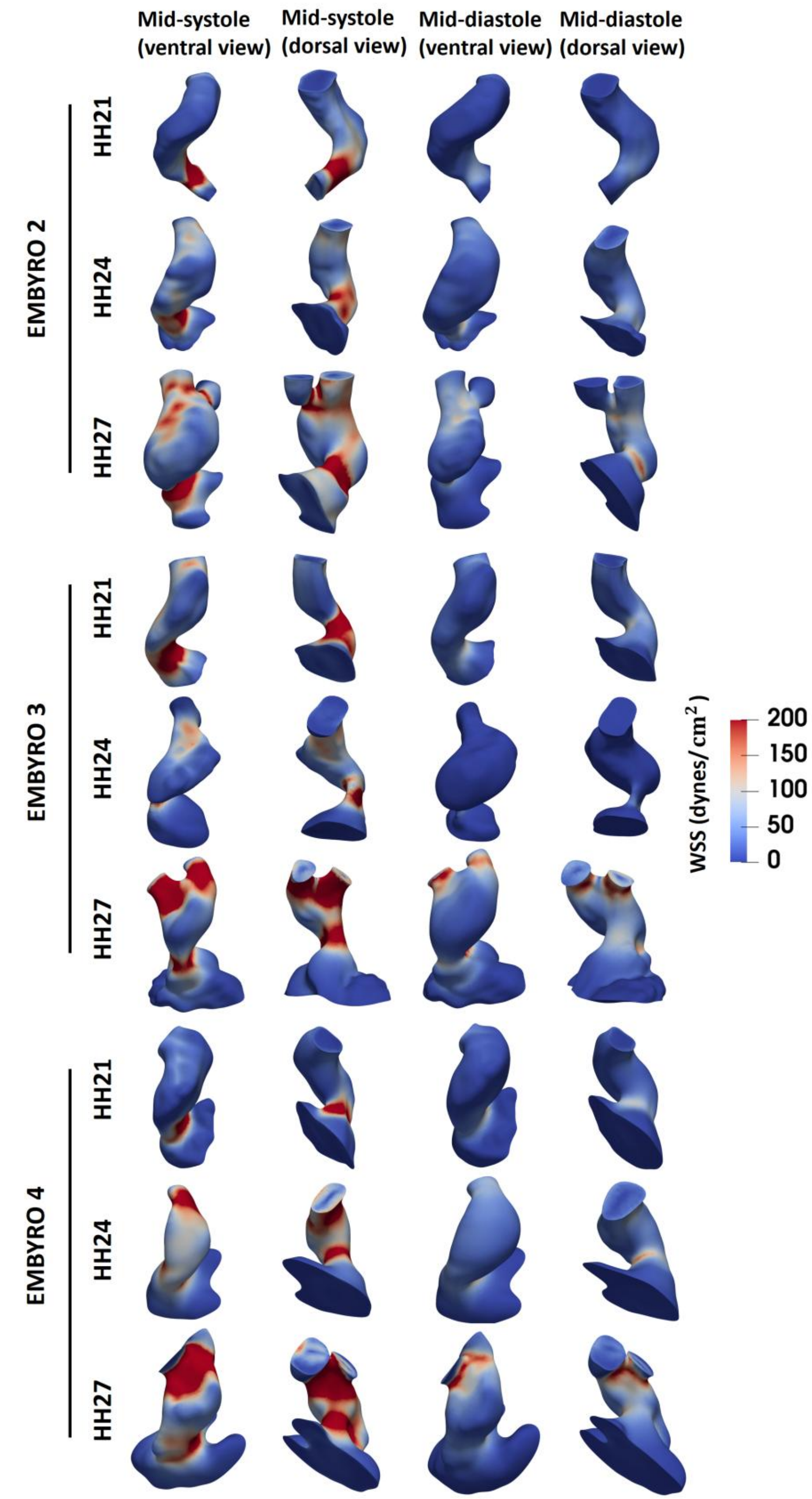

Figure S4: Time- and space-varying WSS magnitudes at different phases of cardiac cycle over development for Embryo 2,3, and 4.

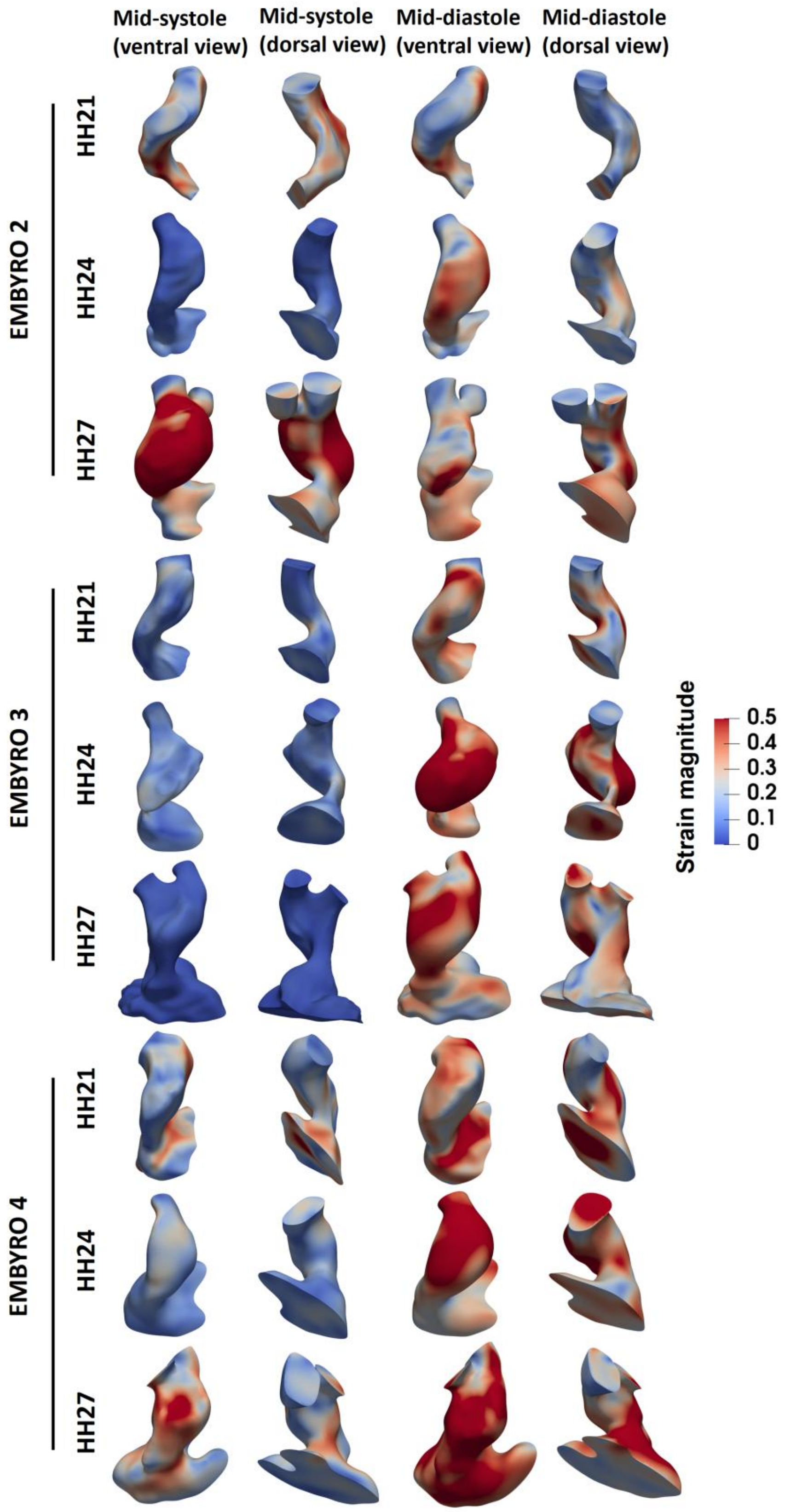

Figure S5: Time- and space-varying strain magnitudes at different phases of cardiac cycle over development for Embryo 2,3, and 4.

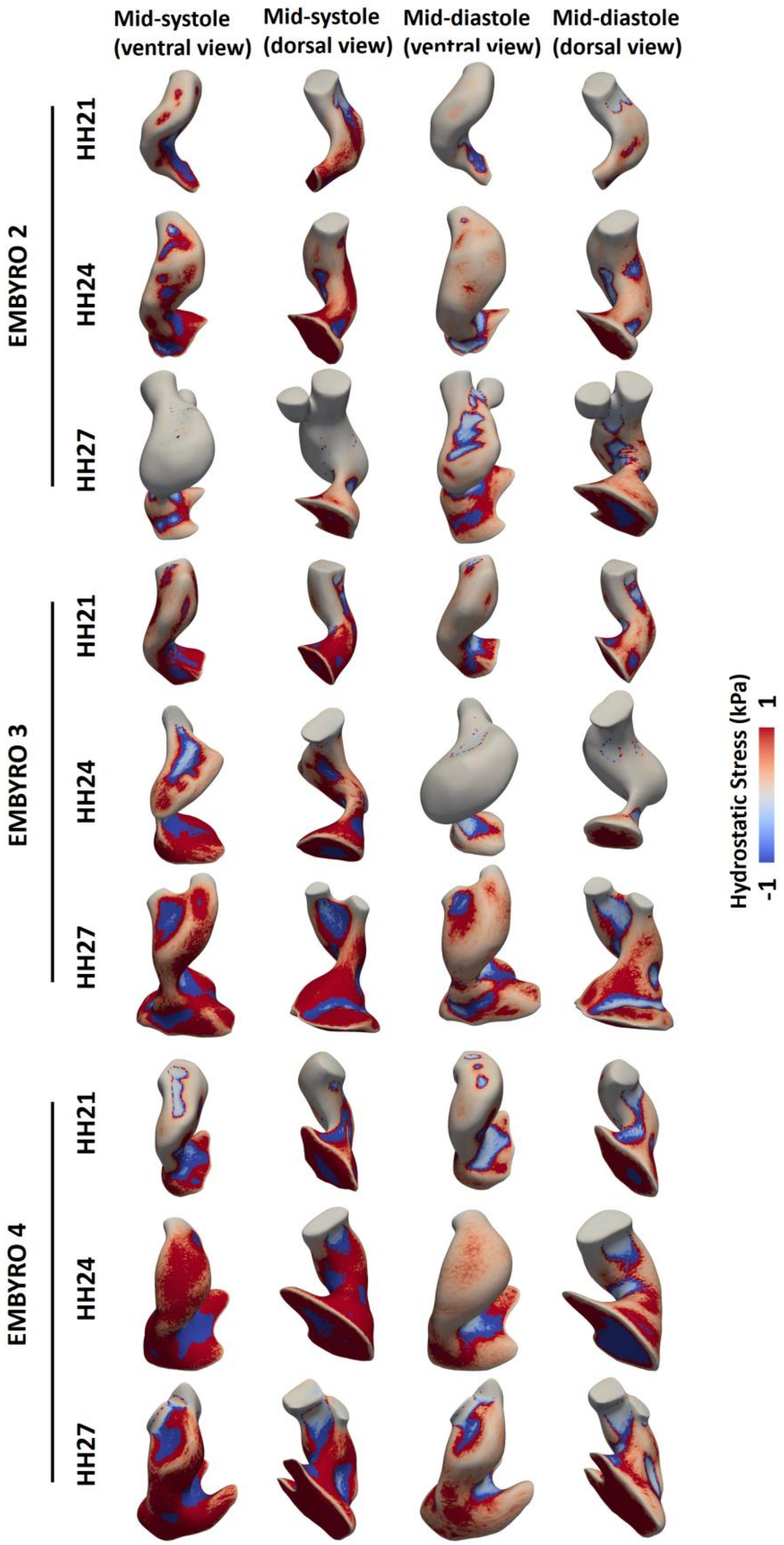

Figure S6: Time- and space-varying hydrostatic wall stress at different phases of cardiac cycle over development for Embryo 2,3, and 4.

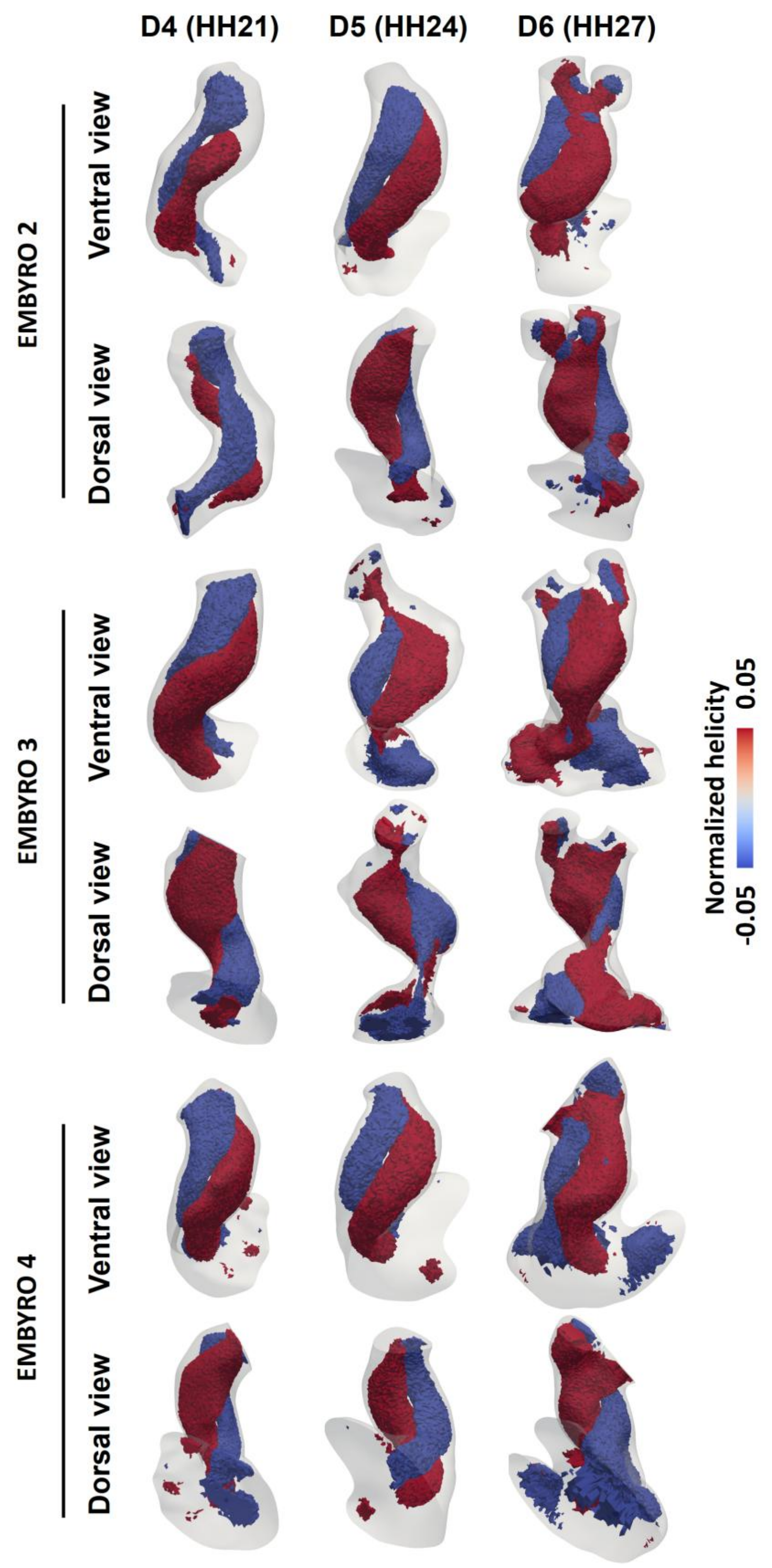

Figure S7: Double-helical flow pattern observed during blood ejection after dO cushions opening, shown in the models of Embryo 2, 3, 4 at different developmental stages.

Supplemental Video Legends

Video S1: Ultrasound cine images showed transition in cushion motions across development at **A)** HH21, **B)** HH24, **C)** HH27. * indicates pO and dO locations.

Video S2: Time- and space-varying WSS magnitudes within a full cardiac cycle over development for Embryo 1 at **A)** HH21, **B)** HH24, **C)** HH27. Arrows indicate blood flow directions. V, ventricle.

Video S3: Time- and space-varying expansive strain from minimum volume within a full cardiac cycle over development for Embryo 1 at **A)** HH21, **B)** HH24, **C)** HH27. Arrows indicate blood flow directions. V, ventricle.
